# Psychophysiological Patterns Related to Success in a Special Operation Selection Course

**DOI:** 10.3389/fphys.2019.00867

**Published:** 2019-07-10

**Authors:** Alberto J. Hormeño-Holgado, Pantelis T. Nikolaidis, Vicente J. Clemente-Suárez

**Affiliations:** ^1^Faculty of Sports Sciences, Universidad Europea de Madrid, Villaviciosa de Odón, Spain; ^2^Studies Centre in Applied Combat (CESCA), Toledo, Spain; ^3^Exercise Physiology Laboratory, Nikaia, Greece; ^4^Grupo de Investigación en Cultura, Educación y Sociedad, Universidad de la Costa, Barranquilla, Colombia

**Keywords:** military, perceived stress scale, combat, anaerobic training, endurance training

## Abstract

Actual theaters of operations require fast actions from special operations units with a high level of readiness and survival. Mission accomplishment depends on their psychological and physiological performance. The aim of the present study was to analyze: (1) the physical parameters related with success in a special operation selection course; and (2) the modifications of the psychological profile of recruits before and after a special operation selection course. Fifty-five male soldiers of the Spanish Army (25.1 ± 5.0 years, 1.8 ± 0.1 cm, 76.8 ± 7.9 kg, 24.4 ± 2.5 kg/m^2^) undertaking a 10-week special operation selection course performed a battery of physiological and psychological tests. Results showed how successful soldiers presented higher leg strength, anaerobic running performance, and cardiovascular response than non-successful soldiers. The psychological values of life engagement test, acceptance and action questionnaire, coping flexibility scale, and perceived stress scale did not present significant differences after the selection course. We can conclude that success in a special operation selection course was related to higher anaerobic and cardiovascular fitness. This special operation selection course did not modify the psychological profile of successful soldiers.

## Introduction

Actual armed conflicts have changed from traditional symmetrical confrontations to an asymmetrical battlefield, characterized by an unstructured and undefined battlefield, where the presence of civilians is becoming more prevalent (Tornero-Aguilera et al., 2017). Those actual theaters of operations require actions to be carried out promptly and briefly with a high level of readiness and survival, sometimes carrying a heavy load, fast movements, tactical parachute jumps or obstacles crossing, being special operations units basic for these new war operations (Tornero-Aguilera et al., 2018). Special operations units are those taking charge of these missions, where their survivability and mission accomplishment depend on their psychological and physiological performance (Tornero-Aguilera and Clemente-Suárez, 2018; Hormeño-Holgado et al., 2019). These situations under extreme conditions have been previously studied in high-demands context as ultraendurance events (Belinchon-deMiguel and Clemente-Suárez, 2018), parachuting (Clemente-Suárez et al., 2017a,b,c), different military combat situations like melee combat (Clemente-Suarez et al., 2018), airmobile protection teams actions (Hormeño-Holgado and Clemente-Suárez, 2019a,b) and in military aircraft pilots (Bustamante-Sánchez et al., 2018, 2019; Hormeño-Holgado and Clemente-Suárez, 2019a,b). During these situations, an increase in sympathetic nervous system, lactic anaerobic metabolism, muscular and cardiovascular response and a decrease in cortical arousal, time perception, rate of perceived exertion and memory were observed (Clemente-Suarez et al., 2018; Delgado-Moreno et al., 2018; Tornero-Aguilera et al., 2018). The constant exposition to these extreme conditions might also lead warfighters to psychologically related disorders, such as anxiety, depression, or post-traumatic stress disorder (Grossman and Siddle, 2008; Hines et al., 2014; Xue et al., 2015). Recent literature supports that previous experience and training influence the psychophysiological responses to these contexts (Tornero-Aguilera and Clemente-Suárez, 2018) and propose specific training models for this context and population as a useful tool to improve the warfighters’ psychophysiological response and operability (Diaz-Manzano et al., 2018a; Curiel-Regueros et al., 2019; Tornero-Aguilera and Clemente-Suárez, 2019).

The psychophysiological profile of a special operations unit must be specific and flexible to prepare their members to deal with any threat that may appear during combat. Previously, physical training has been recognized as essential for readiness (Curiel-Regueros et al., 2019; Tornero-Aguilera and Clemente-Suárez, 2019), whereas an optimal psychological structure is critical to empowering the warfighter with a better response in high-stress conditions (Bellido et al., 2018). Despite its importance, the physiological training patterns of special operations soldiers, as well as the modifications in their psychological profile, have not been determined. Therefore, the aim of the present study was to analyze: (1) the physical parameters related with success in a special operation selection course and (2) the modifications of the psychological profile of recruits during a special operation selection course. The research hypotheses were: (1) aerobic fitness would be related to success in a special operation selection course and (2) a special operation selection course would increase resilience and decrease perceived stress of recruits.

## Methods and Materials

### Participants

Fifty-five male soldiers of the Spanish Army (25.1 ± 5.0 years, 1.8 ± 0.1 cm, 76.8 ± 7.9 kg, 24.4 ± 2.5 kg/m^2^) undertaking a 10-week special operation selection course participated in this study. All participants had 29.1 ± 59.3 months of professional experience at the armed forces, 4.3 ± 7.7 months at the unit, and 0.5 ± 2.5 months of experience of real-life combat in Lebanon, Afghanistan, Bosnia, Kosovo, or Iraq. Prior to participation, the experimental procedures were explained to participants, who provided their voluntary written informed consent in accordance with the Declaration of Helsinki. The headquarters of the unit and the military ethics committee approved the study protocols and procedures.

### Procedure

Before the 10-week special operation selection course, physical tests were performed in order to complete the specific objective (1) before, during, and after the 10-week special operation selection course, psychological tests were administrated to the participants in order to complete the specific objective (2).

In the special operation selection course, participants had to deal with endurance walks, decision-making scenarios under pressure, obstacle courses, group heavy lifting, ground and water survival, maneuvers under fire or room breaching and clearing among others.

#### Physical Tests

The following physical tests were conducted:

1. In lower body muscular strength by means of horizontal jump test, subjects performed a standardized warm-up consisting of 2x10 vertical jumps with 30 seconds of recovery and then, they performed two maximal horizontal jumps as mentioned in a previous report (Clemente-Suárez et al., 2018) and the best attempt was used for the statistical analysis.
2. Anaerobic performance was measured by the time of completion of a 50-m maximal running test.
3. Aerobic performance was measured by a 2000-m maximal running test, which is associated with the maximal aerobic speed measured in an incremental test conducted in laboratory (Coutts et al., 2007). Heart rate (HR) was evaluated by a polar s600 (Polar electro. Finland).
4. Rating of perceived exertion (RPE) by the Borg 6–20 scale was evaluated in both running tests.

#### Psychological Tests

The following psychological tests were administered:

1. The life engagement test (LET) was designed to measure purpose in life, defined in terms of the extent to which a person engages in activities that are personally valued (Scheier et al., 2006).
2. Acceptance and action questionnaire (AAQ-II): measured psychological inflexibility considering it as a transdiagnostic process across psychological disorders (Wolgast, 2014).
3. The coping flexibility scale (CFS): this scale was designed to measure the flexibility in coping with different situations. It refers to the presence of adaptive coping strategies that are associated with better psychological health. This test has 10 items and is answered on a Likert scale ranging from 1 to 4, with 1 = very applicable and 4 = not applicable. Item example: “I am aware of the success or failure of my attempts to deal with stress” (Kato, 2012).
4. Perceived stress scale (PSS): this scale assesses the level of perceived stress in a 1-month period. It is composed of 14 items that are answered in a 5-point Likert scale, where 0 = never and 4 = very often. An example item is “In the last month, how often have you felt that had everything under control?” High scores are related to a higher perception of stress (Cohen et al., 1983).

### Statistical Analysis

To analyze the data, we used the SPSS statistical package (version 22.0; SPSS, Inc., Chicago, III.). Means and standard deviation (SD) were calculated using traditional statistical techniques. Normality and homoscedasticity assumptions were checked with a Kolmogorov–Smirnov test. To analyze differences between pre, during and post special operation selection course samples, a One-way ANOVA for dependent samples was administer, and to analyze differences between successful and non-successful soldiers in the special operation selection course a *t*-test for independent samples was used. The effect size (ES) was tested by Cohen’s *d* [ES = (post-test mean − pre-test mean)/pre-test SD]. The level of significance for all the comparisons was set at *p* < 0.05.

## Results

Results are reported as mean ± SD. Physical variables of successful and non-successful soldiers in the special operation selection course showed that horizontal jump, 50-m performance, and HR in the 2000-m tests where significantly (*p* < 0.05) higher in successful than in non-successful soldiers. The values of weight, RPE in 50 m test, 2000 m test and RPE in 2000 n test between successful and non-successful soldiers did not present significant differences (Table 1).

**Table 1:** Physical variables of successful and non-successful soldiers in the special operation selection course.

Variable	Successful	Non-successful	*t*	*p*	Cohen’s *d*	95% CI of the differences	
						Lower	Upper
Weight (kg)	76.24 ± 13.86	73.14 ± 10.66	−0.634	0.814	−0.22	−12.9086	6.7105
Horizontal jump (cm)	220.20 ± 20.07	189.89 ± 37.34	−3.455	−0.002	−1.51	−47.944	−12.669
50-m test (s)	7.64 ± 0.47	8.31 ± 0.96	3.158	0.001	1.43	0.2442	1.1000
RPE in 50-m test	10.88 ± 2.71	11.89 ± 2.71	1.027	0.844	0.37	−0.968	2.990
2000-m test (min:s)	9:06 ± 0:45	9:38 ± 0:54	1.810	0.492	0.71	−0:03	1:06
HR in 2000-m test (bpm)	163.32 ± 18.37	153.78 ± 26.71	−1.295	0.010	−0.52	−24.345	5.266
RPE in 2000-m test	15.10 ± 1.79	15.89 ± 1.54	1.231	0.526	0.44	−0.502	2.084

*HR, heart rate; RPE, rated perceived exertion*.

The values of the psychological variables measured before, during, and after the special operation selection course (LET, AAQ-II, CFS, and PSS) did not present significant modifications (*p* > 0.05) during the entire selection course (Table 2).

**Table 2:** Psychological variables before, during, and after the special operation selection course.

Variable	M1	M2	M3	M1 vs. M2	M1 vs. M3	M2 vs. M3
LET	27.98 ± 2.21	27.72 ± 2.16	28.18 ± 1.7	0.645	0.459	0.588
AAQ-II	5.82 ± 4.21	6.08 ± 4.34	5.16 ± 4.15	0.521	0.742	1.000
CFS	18.23 ± 4.87	18.11 ± 5.76	18.47 ± 4.87	0.614	0.849	1.000
PSS	19.96 ± 6.42	19.44 ± 7.97	20.77 ± 7.88	0.689	0.598	0.759

*M, momentum of sample; M1, before special operation selection course; M2, middle stage of special operation selection course; M3, after the special operation selection course; LET, life engagement test; AAQ-II, acceptance and action questionnaire; CFS, coping flexibility scale; PSS, perceived stress scale*.

## Discussion

The aim of this study was to analyze: (1) the physical parameters related to success in a special operation selection course and (2) the modifications of the psychological profile of recruits before and after a special operation selection course. The first hypothesis was not compiled since anaerobic fitness was related with success in the special operation selection course, and the second hypothesis was also not complied since no modifications in psychological profile were found.

Successful soldiers in the special operations selection course presented significantly higher leg strength, anaerobic running performance, and cardiovascular response in the aerobic running test than non-successful soldiers. These results highlighted the importance of anaerobic metabolic pathways in line with present researches focused on the psychophysiological response in different combat contexts. In these studies, authors found a large anaerobic metabolism activation measured by the blood lactate concentration in military parachute jumps, close quarter combat, urban combat, and airmobile protection teams (Clemente-Suarez and Robles-Perez, 2013; Delgado-Moreno et al., 2017; Sánchez-Molina et al., 2017; Clemente-Suárez et al., 2017a,b,c; Diaz-Manzano et al., 2018a,b; Hormeño-Holgado et al., 2019). In this line, the higher 2000-m HR of successful soldiers in the present research also showed how the possibility to reach a higher physical effort was an important factor for these special operations units. Previous authors found how elite soldiers differ from non-elite soldiers by the higher cardiovascular and metabolic output in combat simulations (Tornero-Aguilera et al., 2017). The capacity to reach this large organic response led them to comply with their tactical objective, increasing their operability. This fact could explain the differences in HR by the successful and non-successful soldiers of the present research, as well as previous authors found how combat actions are conducted with intensities over the anaerobic threshold, fact that also highlighted the importance of been able to manifest a high cardiovascular response (Clemente-Suárez et al., 2016; Sánchez-Molina et al., 2017; Bustamante-Sánchez et al., 2018, 2019; Hormeño-Holgado et al., 2019). Nerveless, no significant differences were found in the aerobic test performance (2000 m). This is probably because traditional military training is mainly focused on long-running as a core training and assessment method (Poston et al., 2017). Although distance running can improve aerobic endurance, the requirements of actual operations are more related with anaerobic metabolic pathways as current researches showed (Scherr et al., 2013; Poston et al., 2017; Keramidas et al., 2018; Curiel-Regueros et al., 2019; Tornero-Aguilera and Clemente-Suárez, 2019).

Regarding the psychological response, the increase in stress elicits the hyperactivity of the sympathetic nervous system and a transient emotional state characterized by feelings of tension and apprehension observed in other military environments (Delgado-Moreno et al., 2017; Clemente-Suarez et al., 2018; Sánchez-Molina et al., 2018). Maladaptive psychophysiological stress response in combat could negatively affect otherwise soldiers outcomes, supposing a risk for them (Andersen and Gustafsberg, 2016) and supporting the importance of psychological preparation in these units (Clemente-Suarez and Robles-Perez, 2013; Hormeño-Holgado et al., 2019). Psychological tests’ measurements before, during, and after the special operations selection course did not show a significant modification. The psychological screening process before starting the special operations unit could be a determining factor for maintaining the psychological structure required for these high-demand units. This result was in line with previous researches in special operations units where the soldiers’ psychological profile was a variable large stable and with low modification by operative stressors (Tornero-Aguilera et al., 2017). As observed in undergraduate psychology students, coping flexibility and optimism have been previously strongly correlated and considered individually strong predictors of perceived stress and life satisfaction (Reed, 2016). Developing optimism could increase a person’s ability to cope with stress. In a health employee sample, a goodness-of-fit approach to coping flexibility has already been successfully implemented (Iwata and Buka, 2002). Positive coping strategies are found to lead to greater resilience and physical well-being important for military personnel (Chen et al., 2018). Previous researches showed how in special operations units, the psychological profile was a variable large stable and with low modification by operative stressors (Tornero-Aguilera et al., 2017). This could also be based on the soldier’s experience in this kind of environment and the previous screening for these units.

### Practical Application

Analyzing the operative physical demands of soldiers, it was observed that both anaerobic and aerobic metabolism were key factors in missions’ success and operability (Tornero-Aguilera et al., 2017). Both targets could be achieved through HIIT methodology (Diaz-Manzano et al., 2018b; Curiel-Regueros et al., 2019; Tornero-Aguilera and Clemente-Suárez, 2019), showing greater physical outcomes (Clemente-Suárez, 2018), injury prevention (Knapik et al., 2004), and health development (Serrano et al., 2001) than conventional approaches.

## Conclusion

Success in a special operation selection course was related to higher anaerobic performance and cardiovascular fitness. This special operation selection course did not modify the psychological profile of successful soldiers.

## Data Availability

All datasets generated for this study are included in the manuscript and/or the supplementary files.

## Ethics Statement

Universidad Europea de Madrid Ethic Committee, project number: CIPI/18/093.

## Author Contributions

AH-H, PN, and VC-S conceived and conducted the experiment and analyzed the results. All authors wrote and reviewed the manuscript.

### Conflict of Interest Statement

The authors declare that the research was conducted in the absence of any commercial or financial relationships that could be construed as a potential conflict of interest.

The reviewer KMH-M declared a past co-authorship with one of the authors VC-S to the handling editor.
