# Corrigendum: Psychophysiological Patterns Related to Success in a Special Operation Selection Course

**DOI:** 10.3389/fphys.2020.614969

**Published:** 2020-11-03

**Authors:** Alberto J. Hormeño-Holgado, Pantelis T. Nikolaidis, Vicente J. Clemente-Suárez

**Affiliations:** ^1^Faculty of Sports Sciences, Universidad Europea de Madrid, Villaviciosa de Odón, Spain; ^2^Studies Centre in Applied Combat (CESCA), Toledo, Spain; ^3^Exercise Physiology Laboratory, Nikaia, Greece; ^4^Grupo de Investigación en Cultura, Educación y Sociedad, Universidad de la Costa, Barranquilla, Colombia

**Keywords:** military, perceived stress scale, combat, anaerobic training, endurance training

In the original article, the reference for “El-On et al., 2003” was incorrectly written as “El-On, J., Ben-Noun, L., Galitza, Z., and Ohana, N. (2003). Case report: clinical and serological evaluation of echinococcosis of the spine. *Trans. R. Soc. Trop. Med. Hyg*. 97, 567–569. doi: 10.1016/S0035-9203(03)80031-7.” It should be “Kato, T. (2012). Development of the coping flexibility scale: evidence for the coping flexibility hypothesis. *J. Couns. Psychol*. 59, 262–273. doi: 10.1037/a0027770.”

The authors apologize for this error and state that this does not change the scientific conclusions of the article in any way. The original article has been updated.

## References

[B1] Kato T. (2012). Development of the coping flexibility scale: evidence for the coping flexibility hypothesis. J. Couns. Psychol. 59, 262–273. 10.1037/a0027770 22506909

